# Effects of a dissociative drug on fronto-limbic resting-state functional connectivity in individuals with posttraumatic stress disorder: a randomized controlled pilot study

**DOI:** 10.1007/s00213-023-06479-4

**Published:** 2023-10-23

**Authors:** Sarah K. Danböck, Or Duek, Ziv Ben-Zion, Nachshon Korem, Shelley L. Amen, Ben Kelmendi, Frank H. Wilhelm, Ifat Levy, Ilan Harpaz-Rotem

**Affiliations:** 1 Department of Psychology, Paris Lodron University of Salzburg, Salzburg, Austria; 2 Department of Psychiatry, School of Medicine, Yale University, New Haven, CT USA; 3 Department of Psychology, School of Social Sciences, University of Mannheim, Mannheim, Germany; 4 VA Connecticut Healthcare System, Clinical Neurosciences Division, National Center for Posttraumatic Stress Disorder, U.S. Department of Veterans Affairs, West Haven, CT USA; 5 Department of Epidemiology, Biostatistics and Community Health Sciences, School of Public Health, Ben-Gurion University of The Negev, Be’er-Sheva, Israel; 6 Departments of Comparative Medicine and Neuroscience, School of Medicine, Yale University, New Haven, CT USA; 7 Department of Psychology, Yale University, New Haven, CT USA; 8 Wu Tsai Institute, Yale University, New Haven, CT USA

**Keywords:** PTSD, Dissociation, Trauma, Biomarker, Ketamine, Imaging, Connectivity

## Abstract

**Rationale:**

A subanesthetic dose of ketamine, a non-competitive N-methyl-D-aspartate glutamate receptor (NMDAR) antagonist, elicits dissociation in individuals with posttraumatic stress disorder (PTSD), who also often suffer from chronic dissociative symptoms in daily life. These debilitating symptoms have not only been linked to worse PTSD trajectories, but also to increased resting-state functional connectivity (RSFC) between medial prefrontal cortex (mPFC) and amygdala, supporting the conceptualization of dissociation as emotion overmodulation. Yet, as studies were observational, causal evidence is lacking.

**Objectives:**

The present randomized controlled pilot study examines the effect of ketamine, a dissociative drug, on RSFC between mPFC subregions and amygdala in individuals with PTSD.

**Methods:**

Twenty-six individuals with PTSD received either ketamine (0.5mg/kg; *n* = 12) or the control drug midazolam (0.045mg/kg; *n* = 14) during functional magnetic resonance imaging (fMRI). RSFC between amygdala and mPFC subregions, i.e., ventromedial PFC (vmPFC), dorsomedial PFC (dmPFC) and anterior-medial PFC (amPFC), was assessed at baseline and during intravenous drug infusion.

**Results:**

Contrary to pre-registered predictions, ketamine did not promote a greater increase in RSFC between amygdala and mPFC subregions from baseline to infusion compared to midazolam. Instead, ketamine elicited a stronger transient decrease in vmPFC-amygdala RSFC compared to midazolam.

**Conclusions:**

A dissociative drug did not increase fronto-limbic RSFC in individuals with PTSD. These preliminary experimental findings contrast with prior correlative findings and call for further exploration and, potentially, a more differentiated view on the neurobiological underpinning of dissociative phenomena in PTSD.

**Supplementary Information:**

The online version contains supplementary material available at 10.1007/s00213-023-06479-4.

## Introduction

Dissociation is characterized by disruptions in and fragmentation of the usually integrated functions of consciousness, memory, identity, body awareness, and perception of the self and the environment (American Psychiatric Association 2013). A well-established pharmacological manipulation of dissociation is intravenous infusion of ketamine, a non-competitive N-methyl-D-aspartate glutamate receptor (NMDAR) antagonist often coined as “dissociative drug” (Denomme 2018; Ballard and Zarate 2020). After initial observations of altered consciousness and awareness of the self and environment during administration of subanesthetic doses of ketamine in the 1960s (Denomme 2018), many studies in healthy and clinical populations have replicated ketamine’s dissociative effects (e.g., Krystal 1994; Short et al. 2018; Duek et al. 2019; Dehestani et al. 2022). The present study focuses on ketamine administered at 0.5mg/kg over 40 min, a subanesthetic dose and infusion time frequently studied in individuals with psychiatric disorders (Feder et al. 2014, 2021; Short et al. 2018; Duek et al. 2019; Dehestani et al. 2022; Abdallah et al. 2022). Under these conditions, dissociation arises shortly after infusion onset and remits about 120 min later (Feder et al. 2014, 2021; Short et al. 2018; Duek et al. 2019; Dehestani et al. 2022; Abdallah et al. 2022).

Importantly, ketamine-induced dissociation psychometrically resembles chronic dissociative symptoms (Niciu et al. 2018; Mertens and Daniels 2022) experienced by many individuals with PTSD (White et al. 2022). Those posttraumatic dissociative symptoms have not only been linked to higher PTSD severity, chronicity, functional impairment, and suicidality (Stein et al. 2013), prompting the introduction of a dissociative PTSD subtype in the Diagnostic and Statistical Manual of Mental Disorders-Fifth Edition (DSM-5; American Psychiatric Association 2013), but have also been associated with a unique neural profile (Lanius et al. 2010; Harnett and Lebois 2022). While PTSD is usually characterized by *emotion undermodulation* mediated by limbic hyperactivation and decreased prefrontal regulation, the dissociative subtype was characterized by *emotion overmodulation* mediated by increased prefrontal activation and limbic hypoactivation during symptom provocation (Lanius et al. 2010). Beyond task-based activations, the dissociative PTSD subtype was also characterized by a unique resting-state functional connectivity (RSFC) profile (Harnett and Lebois 2022). In line with the *emotion overmodulation* model (Lanius et al. 2010), individuals with the dissociative PTSD subtype displayed increased RSFC between amygdala and prefrontal cortex (PFC) regions in charge of emotional regulation (Nicholson et al. 2015). Moreover, directed connectivity analyses in these individuals supported a predominant “top-down” connectivity, from the ventromedial PFC (vmPFC) to the amygdala, as opposed to a more “bottom-up” connectivity in PTSD individuals without dissociative symptoms (Nicholson et al. 2017). Other studies also yielded differences between PTSD individuals with and without dissociative symptoms in whole-brain seed-based RSFC analyses using various seed regions including insula, bed nucleus of the stria terminalis, cerebellum, periaqueductal gray, vestibular nuclei, pulvinar and superior colliculi (Nicholson et al. 2016; Harricharan et al. 2016, 2017, 2020; Olivé et al. 2018; Rabellino et al. 2018a, b; Terpou et al. 2018). However, as all studies were observational by nature, i.e., relied on group comparisons between individuals with and without dissociative symptoms, it is unclear whether findings are linked to the dissociation phenomenon itself or to other differences between groups like prior traumatic exposure or comorbidity (Hansen et al. 2017). Hence, experimentally inducing dissociation in individuals with PTSD is indispensable to draw conclusions about the specificity of RSFC alterations for dissociation in this population.

Here, we examined effects of ketamine, a dissociative drug, on RSFC in individuals with PTSD. Controlling for potential effects of chronic dissociative symptoms on state dissociation (Leonard et al. 1999; Mello et al. 2021), our sample mainly consisted of individuals with PTSD without chronic dissociative symptoms. Participants received either ketamine (0.5mg/kg over 40 min), a drug which has previously been shown to elicit dissociation in this population at this dose and infusion time (Feder et al. 2014, 2021; Abdallah et al. 2022), or the control drug midazolam (0.045mg/kg over 40 min), a benzodiazepine which has previously been used in this population at this dose and infusion time to account for subjective effects of ketamine other than dissociation (e.g., blurred vision, dry mouth, fatigue, and headache) and preserve blinding (Feder et al. 2014, 2021). RSFC was assessed at baseline and during intravenous drug infusion.

As the fronto-limbic system has been deemed important for trauma-related dissociation during symptom provocation and at rest (Lanius et al. 2010; Nicholson et al. 2015, 2017), we *a priori* restricted our analyses to the link between amygdala and mPFC (see our pre-registration: 10.17605/OSF.IO/3RFEG). Previous studies varied in their definition of examined amygdala and mPFC (sub-)regions. Hence, we decided to use non-overlapping bilateral functional parcels based on meta-analytic coactivation derived from over 10,000 studies (de la Vega et al. 2016; Chang et al. 2021) for amygdala, vmPFC, dorsomedial PFC (dmPFC), and anterior-medial PFC (amPFC). We tentatively hypothesized that ketamine would promote a stronger increase in RSFC between amygdala and mPFC subregions from baseline to infusion than midazolam.

## Methods

### Participants

Twenty-eight participants with PTSD according to the Clinician-Administered PTSD Scale for DSM-5 (Weathers et al. 2018) and currently not engaged in trauma-focused therapy were randomized to either ketamine or midazolam infusion as part of a registered double-blind clinical trial described elsewhere (Duek et al. 2023). Of those, 26 participants completed the ketamine (*n* = 12)/ midazolam (*n* = 14) infusion during functional MRI (fMRI) constituting the sample for the current secondary analyses (for a CONSORT flow diagram see Supplements, Figure S1). Sample characteristics are displayed in Table 1.

Exclusion criteria included lifetime bipolar disorder, borderline personality disorder, obsessive-compulsive disorder, schizophrenia or schizoaffective disorder, or current psychotic symptoms assessed by the Structured Clinical Interview for DSM-IV (First et al. 1995). Moreover, no participants with dementia, current suicide risk, moderate-to-high severity of substance use disorder (in the three months prior to randomization), history of mild-to-severe traumatic brain injury, or acute medical illness were included in the trial.

**Table 1:** Sample characteristics

	Ketamine (*n* = 12)	Midazolam (*n* = 14)	Group comparison
Female, *no. (%)*	4 (33%)	7 (50%)	*p* = .453
Age in years, *M (SD)*	38.00 (10.35)	36.21 (10.81)	*p* = .671
CAPS, PTSD symptom score (0–80), *M (SD)*	42.33 (11.10)	39.50 (6.55)	*p* = .448
CAPS, dissociation symptom score (0–8), *M (SD)*	0.83 (1.75)	1 (1.57)	*p* = .802
CAPS, dissociative subtype, *no. (%)*	2 (17%)	4 (28%)	*p* = .652
Framewise displacement during infusion, *M (SD)*	0.24 (0.11)	0.40 (0.29)	*p* = .072

Welch *t*-tests and Fisher’s exact tests revealed no differences between ketamine and midazolam groups. Abbreviations: *CAPS* Clinician Administered PTSD Scale

The authors assert that all procedures contributing to this work comply with the ethical standards of the relevant national and institutional committees on human experimentation and with the Helsinki Declaration of 1975, as revised in 2008. All procedures involving human subjects were approved by the Yale University Institutional Review Board (IRB). Written informed consent was obtained from all participants.

### Procedure

Embedded in a larger study protocol described elsewhere (Duek et al. 2023), participants completed a 10-min baseline resting-state scan (9:40 min of actual acquisition) and a 40-min resting-state scan during ketamine (0.5mg/kg) or midazolam (0.045mg/kg) infusion. Overall, analyses were performed on 10-min time segments, to increase comparability between baseline and infusion data and to take into account dynamic changes during infusion.

Due to technical problems, the infusion rate was higher and thus infusion was completed earlier for four subjects (ketamine: *n* = 3, midazolam: *n* = 1) with a minimum infusion duration of 30 min. Moreover, infusion started approximately 7 min before the infusion resting-state scan for one subject (midazolam: *n* = 1), which is why infusion data was missing for the first 7 min of infusion for this subject.

To deal with these divergences, we opted for an approach preserving as much data as possible while ensuring comparability of infusion data between subjects: using the first, the middle and the last 10-min segment of each participant’s *individual* infusion time and only excluding those segments for which data was partially not obtained (i.e., the first 10 min of the one subject of whom a substantial amount of the first 10 min of infusion were not recorded).

### MRI data acquisition and preprocessing

MRI data were collected with a Siemens 3T Prisma scanner with a 32-channel receiver array head coil. High-resolution structural images were acquired by Magnetization-Prepared Rapid Gradient-Echo (MPRAGE) imaging (TR = 1.9 s, TE = 2.77 ms, TI = 900 ms, flip angle = 9°, 176 sagittal slices, voxel size = 1 ×1 × 1 mm, 256 × 256 matrix in a 256-mm FOV). Functional MRI scans were acquired using a multi-band Echo-planar Imaging (EPI) sequence (multi-band factor = 4, TR = 1000 ms, TE = 30 ms, flip angle = 60°, voxel size = 2 × 2 × 2 mm^3^, 60 2-mm-thick slices, in-plane resolution = 2 × 2 mm^2^, FOV = 220 mm).

After preprocessing using *FMRIPrep* version 1.5.8 (Esteban et al. 2019), we smoothed the data (fwhm = 6 mm) and performed voxelwise denoising using nltools (https://github.com/cosanlab/nltools). Specifically, we regressed out the following parameters: average cerebral spinal fluid activity, white matter signal, framewise displacement, six rotation and translations parameters, their squares, derivatives, and squared derivatives, dummy coded spikes identified from global signal and frame differencing outliers (defined as greater than three SDs above the mean), and linear and quadratic trends. We excluded the first five TRs of both the baseline and the infusion sequence and performed the analyses on the remaining time series.

### Parcellation and RSFC estimates

We extracted vmPFC, dmPFC, amPFC, and amygdala time series using a set of non-overlapping, bilateral parcels (https://identifiers.org/neurovault.collection:2099) that have been created based on meta-analytic coactivation in over 10,000 published studies available in the Neurosynth database (de la Vega et al. 2016; Chang et al. 2021).

For statistical analyses, we computed (static) RSFC estimates per segment (baseline; first, middle, and last 10 min of infusion). Specifically, we calculated Spearman correlations between vmPFC-amygdala, dmPFC-amygdala, and amPFC-amygdala time series for all segments of interest and standardized them using a Fisher z-transformation.

For descriptive purposes, we also computed and plotted timepoint-by-timepoint (i.e., dynamic) RSFC estimates using a computational approach developed by Owen et al. (2021). We used the python-based toolbox *timecorr* provided by the authors and a Laplace kernel with a width of 20 which has demonstrated good performance in detecting true correlations across 100 synthetic datasets for a variety of time-dependent correlation changes (e.g., stable correlations over time, smoothly varying correlations, event-based varying correlations; Owen et al. 2021).

### Statistical analyses

Using the Stan-based package *brms* (Bürkner 2017; Carpenter et al. 2017) in R 4.0.3 (R Core Team 2020), we computed Bayesian multilevel regression models to assess whether ketamine causes a stronger increase in RSFC from baseline to infusion between the amygdala and mPFC regions than midazolam. We calculated three separate models with (1) vmPFC-amygdala, (2) dmPFC-amygdala, and (3) amPFC-amygdala RSFC fitted with Gaussian distributions as outcomes. As predictors, we entered group and segment as dummy coded variables (group: midazolam = 0, ketamine = 1; segment: baseline = 0, first 10 min of infusion = 1, middle 10 min of infusion = 2, last 10 min of infusion = 3), as well as the interaction between group and segment. Segment was entered as a categorical variable as descriptive inspection of RSFC estimates denoted a nonlinear development of RSFC over time. We accounted for the four repeated measurements per subject by including a random intercept into each model.

We report regression coefficients (*b*s) and, as recommended (Kruschke 2014; McElreath 2020), 89% credible intervals (CIs), i.e., Bayesian confidence intervals, for group differences in RSFC at baseline and for group × segment interactions (i.e., group differences in RSFC changes from baseline to the first, middle, and last 10 min of infusion). Additionally, we report the posterior probability of each coefficient being greater (PP_*b*>0_) and smaller (PP_*b*<0_) than zero, i.e., the percentage of posterior draws being greater/smaller than zero. Effects were considered significantly different from zero if the estimate’s 89%CIs did not include zero. For significant interactions, we also report *b*s and 89%CIs for within-group changes from baseline to the respective infusion segment and for within-segment differences between groups.

We used weakly or non-informative default priors of brms whose influence on results is negligible (Bürkner 2017, 2018). All Bayesian multilevel regression models converged as indicated by common algorithms-agnostic (Vehtari et al. 2021) and algorithm-specific diagnostics (Betancourt 2017). There were no divergent transitions (*Rhat* < 1.01 and *ESS* > 400) for all relevant parameters.

## Results

### Did ketamine increase vmPFC-amygdala RSFC?

**Fig. 1:**
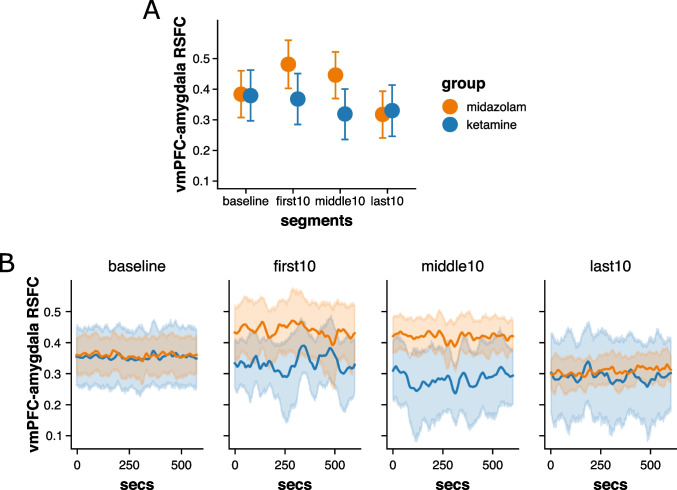
**A** Fitted values of a Bayesian multilevel regression model predicting (static) vmPFC-amygdala RSFC by group (ketamine vs. midazolam) and segment (baseline; first, middle, and last 10 min of drug infusion). Vertical lines represent 89%CIs. **B** Timepoint-by-timepoint (dynamic) vmPFC-amygdala RSFC

Effects of group and segment on vmPFC-amygdala RSFC are illustrated in Fig. 1. Groups did not differ in vmPFC-amygdala RSFC during baseline (*b* = 0.00, 89%CI = [−0.11, 0.11], PP_*b*>0_ = 51%, PP_*b*<0_ = 49%). Contrary to our predictions, our data showed no above-threshold evidence for group differences in the change of vmPFC-amygdala connectivity from baseline to the first 10 min of infusion (*b* = −0.12, 89%CI = [−0.25, 0.00], PP_*b*>0_ = 6%, PP_*b*<0_ = 94%) and the last 10 min of infusion (*b* = 0.01, 89%CI = [−0.11, 0.14], PP_*b*>0_ = 57%, PP_*b*<0_ = 43%). However, ketamine was associated with a larger reduction in vmPFC-amygdala RSFC from baseline to the middle 10 min of infusion compared to midazolam (*b* = −0.14, 89%CI = [−0.26, −0.01], PP_*b*>0_ = 4%, PP_*b*<0_ = 96%). Specifically, while ketamine and midazolam did not differ in vmPFC-amygdala RSFC at baseline (*b* = 0.00, 89%CI = [−0.11, 0.11], PP_*b*>0_ = 51%, PP_*b*<0_ = 49%), ketamine was associated with lower vmPFC-amygdala RSFC than midazolam during the middle 10 min of infusion (*b* = −0.13, 89%CI = [−0.24, −0.02], PP_*b*>0_ = 3%, PP_*b*<0_ = 97%). Within-group changes from baseline to the middle 10 min of infusion did not reach significance (ketamine: *b* = −0.07, 89%CI = [−0.16, 0.02], PP_*b*>0_ = 12%, PP_*b*<0_ = 88%; midazolam: *b* = 0.07, 89%CI = [−0.02, 0.15], PP_*b*>0_ = 90%, PP_*b*<0_ = 10%).

### Did ketamine increase dmPFC-amygdala RSFC?

Groups did not differ in dmPFC-amygdala RSFC during baseline (*b* = 0.04, 89%CI = [−0.09, 0.18], PP_*b*>0_ = 71%, PP_*b*<0_ = 29%). Contrary to our predictions, our data showed no above-threshold evidence for group differences in the change of dmPFC-amygdala RSFC from baseline to the first (*b* = −0.11, 89%CI = [−0.26, 0.05], PP_*b*>0_ = 13%, PP_*b*<0_ = 87%), the middle (*b* = −0.10, 89%CI = [−0.25, 0.05], PP_*b*>0_ = 13%, PP_*b*<0_ = 87%), and the last (*b* = 0.02, 89%CI = [−0.13, 0.17], PP_*b*>0_ = 59%, PP_*b*<0_ = 41%) 10 min of infusion (see Supplements, Figure S2).

### Did ketamine increase amPFC-amygdala RSFC?

Groups did not differ in amPFC-amygdala RSFC during baseline (*b* = −0.03 , 89%CI = [−0.19, 0.12], PP_*b*>0_ = 36%, PP_*b*<0_ = 64%). Contrary to our predictions, our data showed no above-threshold evidence for group differences in the change of vmPFC-amygdala RSFC from baseline to the first (*b* = 0.07, 89%CI = [−0.06, 0.19], PP_*b*>0_ = 80%, PP_*b*<0_ = 20%), the middle (*b* = −0.04, 89%CI = [−0.17, 0.08], PP_*b*>0_ = 29%, PP_*b*<0_ = 71%), and the last (*b* = 0.09, 89%CI = [−0.04, 0.22], PP_*b*>0_ = 86%, PP_*b*<0_ = 14%) 10 min of infusion (see Supplements, Figure S3).

## Discussion

The present randomized-controlled pilot study examined effects of ketamine, a dissociation-inducing drug (Feder et al. 2014, 2021; Abdallah et al. 2022), on mPFC-amygdala RSFC in individuals with PTSD. Contrary to our pre-registered hypotheses, individuals who received ketamine did not show a stronger increase in RSFC between amygdala and mPFC subregions from baseline to infusion than individuals who received the control drug midazolam. Instead, our data suggest that ketamine even promoted a greater decrease in vmPFC-amygdala RSFC from baseline to the middle 10 min of infusion compared to midazolam. These preliminary experimental findings contrast with previous theoretical and (correlative) empirical work on the association between dissociation and fronto-limbic RSFC in PTSD (Lanius et al. 2010, 2018; Nicholson et al. 2015, 2017) and call for further exploration, and potentially, a more differentiated view.

Initial observations of increased (top-down) fronto-limbic RSFC in individuals with the dissociative subtype of PTSD (Nicholson et al. 2015, 2017) have supported the idea that dissociation can be conceptualized as enhanced downregulation of negative emotions, i.e., *emotion overmodulation* (Lanius et al. 2010, 2018). However, a recent large study (*N* = 145) did not link persistent dissociation two weeks post trauma to fronto-limbic RSFC (Lebois et al. 2022a). Consistent with these findings, we did not observe increased fronto-limbic RSFC during infusion of a dissociative drug in individuals with PTSD. Instead, our study even linked dissociative drug infusion to a greater transient decrease in fronto-limbic RSFC. This decrease was observed specifically for the vmPFC, a region substantially involved in implicit emotion regulation, i.e., emotion regulation automatically evoked by a stimulus, running without conscious monitoring, and potentially happening without insight and awareness (e.g., inhibition of fear; Etkin et al. 2015). However, it was not observed in two other regions of the mPFC: the dmPFC, a region linked to more explicit emotion regulation strategies (e.g., reappraisal; Buhle et al. 2014; Etkin et al. 2015), and the amPFC, a region associated with evaluative judgment and self-referential processes (Zysset et al. 2003). The observed decrease in vmPFC-amygdala coupling might thus denote that acute dissociation can, under specific circumstances, be coupled with *deficient implicit emotion regulation*, for instance, with deficient fear inhibition. The observed decoupling seems to be strongest in the middle 10 min of infusion, i.e., starting 10 to 15 min after infusion onset. Previous ketamine infusion studies reported the experience of dissociation within 30 min after infusion onset (Abdallah et al. 2022) which is why, in our case, in the absence of dissociation rating data, it is unclear whether the observed decoupling might precede or accompany ketamine-induced dissociation experience.

By pharmacologically manipulating dissociation, our study adds a new angle to the understanding of the link between dissociation and fronto-limbic RSFC in PTSD. However, as our study extends previous work in various aspects, we cannot yet determine why exactly our results deviate from previous findings. One essential advantage differentiating the current from previous studies is the study design. Previous correlative findings (Nicholson et al. 2015, 2017) might have been driven by shared etiology (e.g., early lifetime adversities) of dissociation and maladaptive emotion regulation strategies like emotional suppression (Hansen et al. 2017; Gruhn and Compas 2020) and the link between emotional suppression and increased fronto-limbic RSFC (Picó-Pérez et al. 2018). Moreover, previous studies (Nicholson et al. 2015, 2017) might as well have captured a RSFC pattern resulting from the chronic experience of dissociative symptoms, i.e., the repeated and prolonged occurrence of dissociative symptoms for at least one month, which defines the dissociative PTSD subtype (American Psychiatric Association 2013). The RSFC under this condition does not necessarily resemble the RSFC pattern during acute dissociation. In contrast, our experimental findings could have captured a transient connectivity pattern causally linked to dissociation itself as it unfolds. To examine whether our findings denote a causal relation between decreased fronto-limbic connectivity and dissociation (and not our specific dissociation induction method), future studies might employ other dissociation induction methods like mirror gazing (Shin et al. 2019), hypnosis (Röder et al. 2007), or induced-catalepsy (Hagenaars et al. 2008) and try to weigh in our findings.

This study also differs from others in the circumstances under which dissociation was observed. Here, we employed a dissociative drug to examine RSFC alterations independently from circumstances accompanying naturally occurring dissociation. In contrast, in previous observational studies (Nicholson et al. 2015, 2017; Lebois et al. 2022a), real-life triggers of dissociation, like aversive (trauma-related) stimuli, cognitive overstimulation, and tiredness (Vancappel et al. 2022), might have driven dissociative responding. As those real-life triggers may themselves affect the fronto-limbic system (Robinson et al. 2012), they might account for heterogenous findings on fronto-limbic connectivity during dissociation. Future studies might therefore compare effects of artificial pharmacological and behavioral manipulations of dissociation (Röder et al. 2007; Hagenaars et al. 2008; Shin et al. 2019) to effects of real-life triggers of dissociation (Vancappel et al. 2022) on fronto-limbic coupling. If fronto-limbic connectivity turns out to be differentially affected by the circumstances under which dissociation emerges, dissociation might be conceptualized independently from its complex relationship with emotion and emotion regulation.

Last, it could also be that diverging findings are related to different neurotransmitter systems involved in ketamine-induced and naturally occurring dissociation. As Salvia divinorum, an opioid receptor agonist, has been shown to produce an altered state of consciousness similar to dissociative symptoms (Addy et al. 2015), it has been argued that naturally occurring dissociation could be mediated by the opioid system (Lanius et al. 2018). In contrast, ketamine-induced dissociation might, similarly to ketamine-induced psychotic symptoms (Corlett et al. 2011), be mediated by glutamatergic dysfunction. To examine pharmacological models of trauma-related dissociation, subjective qualities of ketamine/opioid-induced dissociation and naturally occurring dissociation need to be compared. Initial findings indicate that dissociative states induced by ketamine and other NMDAR antagonists (Niciu et al. 2018; Piazza et al. 2022) psychometrically resemble dissociative states experienced by individuals with trauma-related psychopathology (Mertens and Daniels 2022). However, ketamine-induced dissociation seems to be less intense (Rodrigues et al. 2020; Feder et al. 2021) than real-life dissociation in trauma-exposed populations (Mertens et al. 2022), and qualitative interviews suggest that ketamine’s psychoactive effects might not be fully captured by standard dissociation measures (van Schalkwyk et al. 2018). Hence, in sum, there is some, albeit preliminary, evidence supporting a glutamatergic model of dissociation, while the field is still awaiting investigations on the opioid model.

Altogether, evidence on the relationship between dissociation and fronto-limbic RSFC is mixed, including positive (Nicholson et al. 2015, 2017), no (Lebois et al. 2022a; the present study), and negative associations (the present study). Interestingly, this picture converges with recent studies examining limbic activation during emotional tasks (Mertens et al. 2022; Lebois et al. 2022a; Danböck et al. 2023) and not being able to replicate the previously shown link between dissociation and decreased limbic activation (Lanius et al. 2010). Together, recent findings might imply that alterations in the fronto-limbic circuitry are highly context-dependent (Lebois et al. 2022a) which limits their potential as neural markers of trauma-related dissociation. However, it is worth noting that, while findings regarding fronto-limbic circuitry and limbic activation appear to be quite inconclusive, evidence for the general involvement of prefrontal regions in dissociation seems to accumulate (as also reviewed by Roydeva and Reinders 2021). Interestingly, this also converges with initial studies pointing towards effects of ketamine, a dissociation-inducing-drug, on prefrontal global functional connectivity (Abdallah et al. 2017, 2018; Castillo et al. 2023; but see also Kraus et al. 2020), stressing the potential of an in-depth exploration of prefrontal alterations during acute dissociative states. In a similar vein, recent work has already started to explore the relationship of dissociation with alterations in neural networks related to consciousness, awareness of the bodily self, proprioception, and interoceptive awareness (Lanius et al. 2018; Lebois et al. 2021, 2022a, b; Wolf et al. 2023) which might inform an updated conceptualization of dissociation in PTSD.

Despite the promising nature of the current preliminary findings, several limitations of the current work should be noted. First, we did not assess dissociation experience during or directly after the infusion taking place in the fMRI. However, a large body of studies has documented ketamine’s dissociative effect in healthy and clinical populations (Krystal 1994; Short et al. 2018; Duek et al. 2019; Dehestani et al. 2022) with many studies employing a similar ketamine dose and infusion time (i.e., 0.5mg/kg over 40min) in similar populations (i.e., individuals with PTSD and/or depression; Feder et al. 2014, 2021; Short et al. 2018; Abdallah et al. 2022). Nevertheless, future studies might include dissociation ratings at 10, 20, and 30 min after infusion onset to determine the temporal dynamics of RSFC alterations and level of reported dissociation and explore alternative explanations for the present findings. Second, we chose midazolam, an active psychotropic drug as control condition to account for unspecific behavioral effects of ketamine (e.g., blurred vision, drymouth, fatigue, and headache) and preserve blinding (Feder et al. 2014, 2021). This limits our conclusions to the relative effects of ketamine and midazolam. Based on the present data (i.e., the respective posterior probabilities), it appears likely that both ketamine and midazolam have contributed to the present findings, with only the contribution of ketamine being the focus of the present study. Future studies might follow-up on this specific effect by comparing ketamine infusion to an inert control condition. Third, we did not assess blinding. However, we deem it unlikely that the subject’s potential capacity to guess which drug they were assigned to affects our RSFC findings. Fourth, while the infusion resting-state data were collected with eyes closed (as recommended for psychedelic resting-state neuroimaging; McCulloch et al. 2022), baseline data were acquired with eyes open. However, differences between eyes open and eyes closed conditions in RSFC have mainly been found for visual, auditory, and sensorimotor networks (Agcaoglu et al. 2019) which were not examined within the present study. Nevertheless, we encourage future studies to also collect baseline data with eyes closed to rule out interactions between eye closure and drug type. Fifth, as our analysis was specific and limited to the three hypotheses tested, we did not employ an additional correction for multiple comparisons. Last, due to the high costs and intricate complexities of pharmacological fMRI studies in clinical populations, our sample size was relatively small. Nevertheless, as also recently pointed out by Marek et al. (2022), small-sample neuroimaging should not be underestimated in the context of complex and hard to conduct studies and experimental interventions, as efficient discovery might involve numerous smaller studies using rigorous methods and scaling up promising results to larger samples. In this vein, future work might weigh in the findings of our initial discovery study in further smaller and larger studies in other subsamples of the PTSD population.

## Conclusion

To the best of our knowledge, the present pilot study was the first randomized-controlled study examining effects of ketamine, a dissociative drug, on fronto-limbic RSFC in individuals with PTSD. Altogether, our findings suggest that dissociation may not necessarily include downregulation of negative emotions mediated by fronto-limbic hyperconnectivity (*emotion overmodulation*). Instead, it might, in some instances, also include *deficient emotion regulation* mediated by fronto-limbic hypoconnectivity. Diverging findings might result from different designs, different circumstances under which dissociation arises, or from different neurotransmitter-systems involved. Future studies might therefore expand on the observational studies examining the dissociative subtype of PTSD and compare a broad range of experimental dissociation induction methods along with dissociation ratings to provide novel insights into the mechanisms and boundary conditions of dissociation in PTSD.

### Supplementary information

ESM 1 (PDF 362 KB)

## Data Availability

Data and analysis code are available on request.
