# Supplementary material for: Effects of a dissociative drug on fronto-limbic resting-state functional connectivity in individuals with posttraumatic stress disorder: a randomized controlled pilot study
Source: Psychopharmacology (Berl). 2023 Oct 23;241(2):243–52. doi: 10.1007/s00213-023-06479-4 (PMC10806226; doi:10.1007/s00213-023-06479-4)
Supplement: Supplementary file 1 — (PDF 362 KB) [file 213_2023_6479_MOESM1_ESM.pdf]

Psychopharmacology

Effects of a dissociative drug on fronto-limbic resting-state functional connectivity in individuals with posttraumatic stress disorder: a randomized controlled pilot study

Supplemental material

Sarah K. Danboeck, Or Duek, Ziv Ben-Zion, Nachshon Korem, Shelley L. Amen, Ben Kelmendi, Frank H. Wilhelm, Ifat Levy, & Ilan Harpaz-Rotem

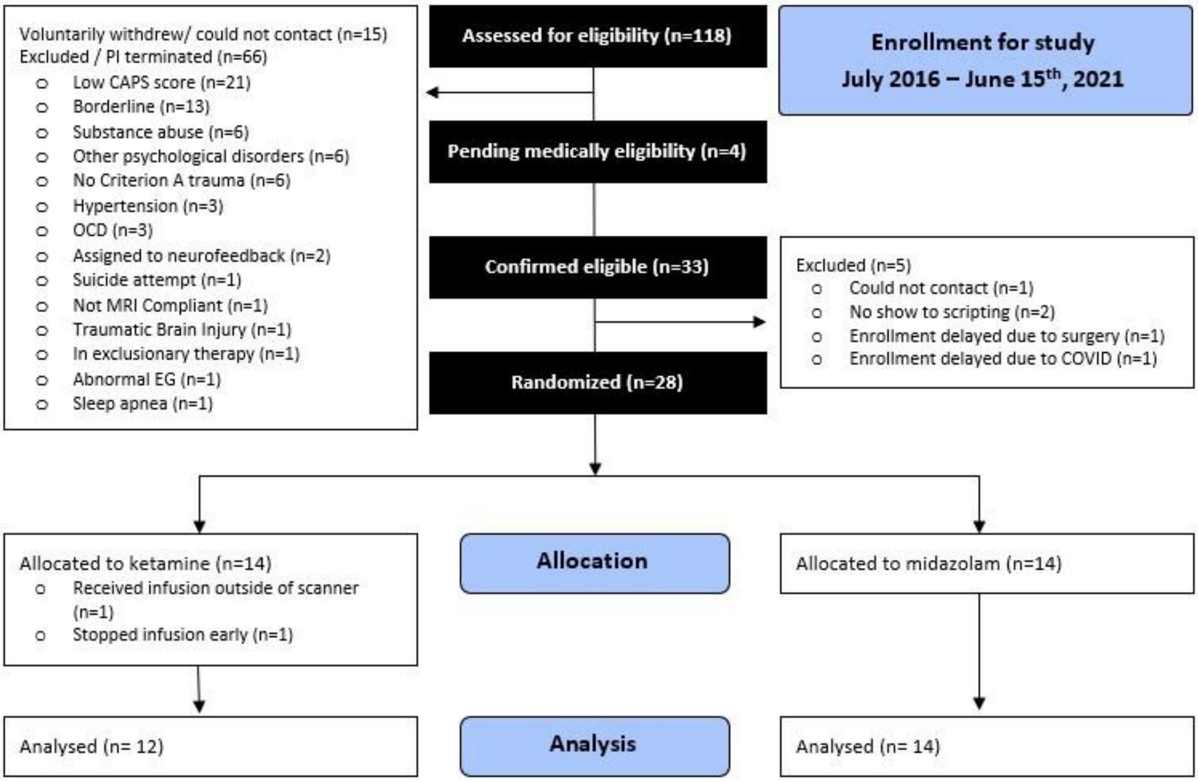

Figure S1. CONSORT flow diagram. The current paper examines data of the infusion session of a larger clinical trial. For a description of the full clinical trial see Duek et al (2023).

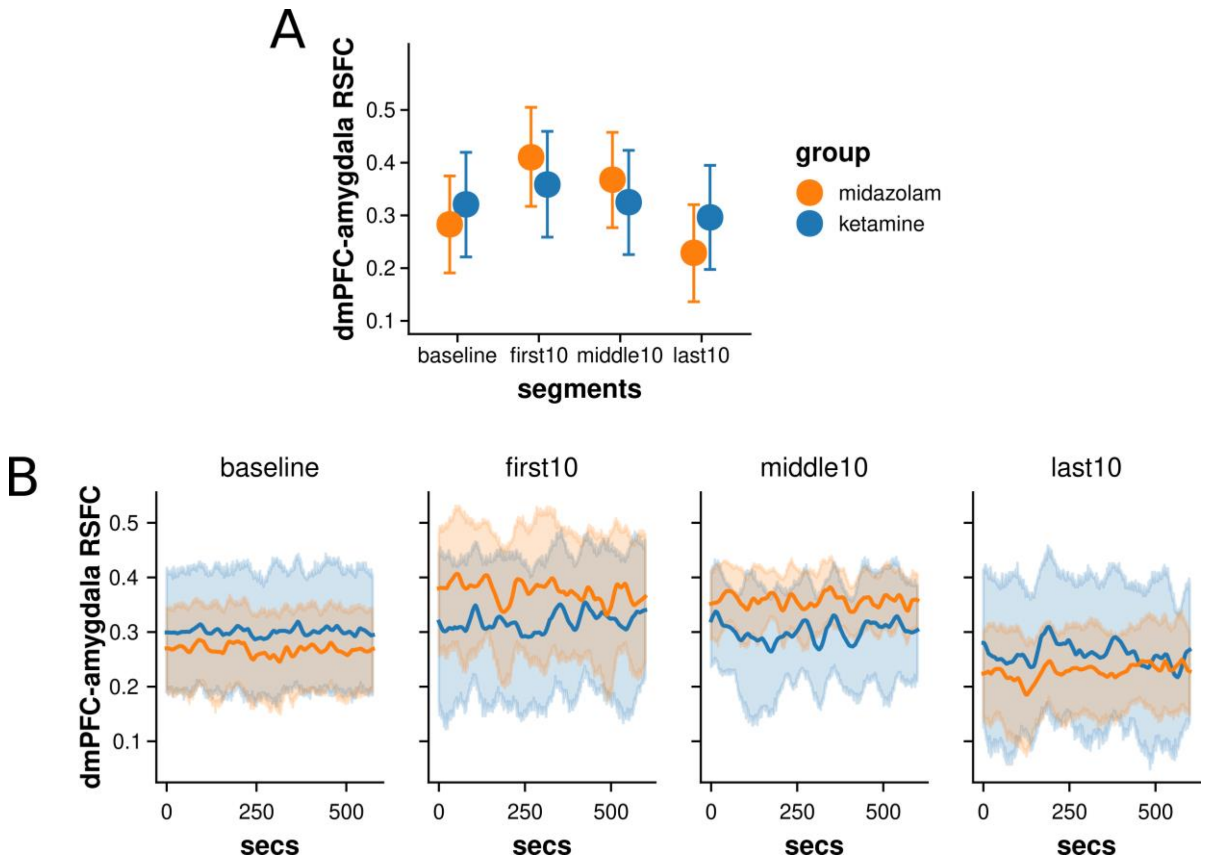

Figure S2. (A) Fitted values of a Bayesian multilevel regression model predicting (static) dmPFC-amygdala RSFC by group (ketamine vs. midazolam) and segment (baseline; first, middle, and last 10-minutes of drug infusion). Vertical lines represent 89%CIs. (B) Timepoint-by-timepoint (dynamic) dmPFC-amygdala RSFC.

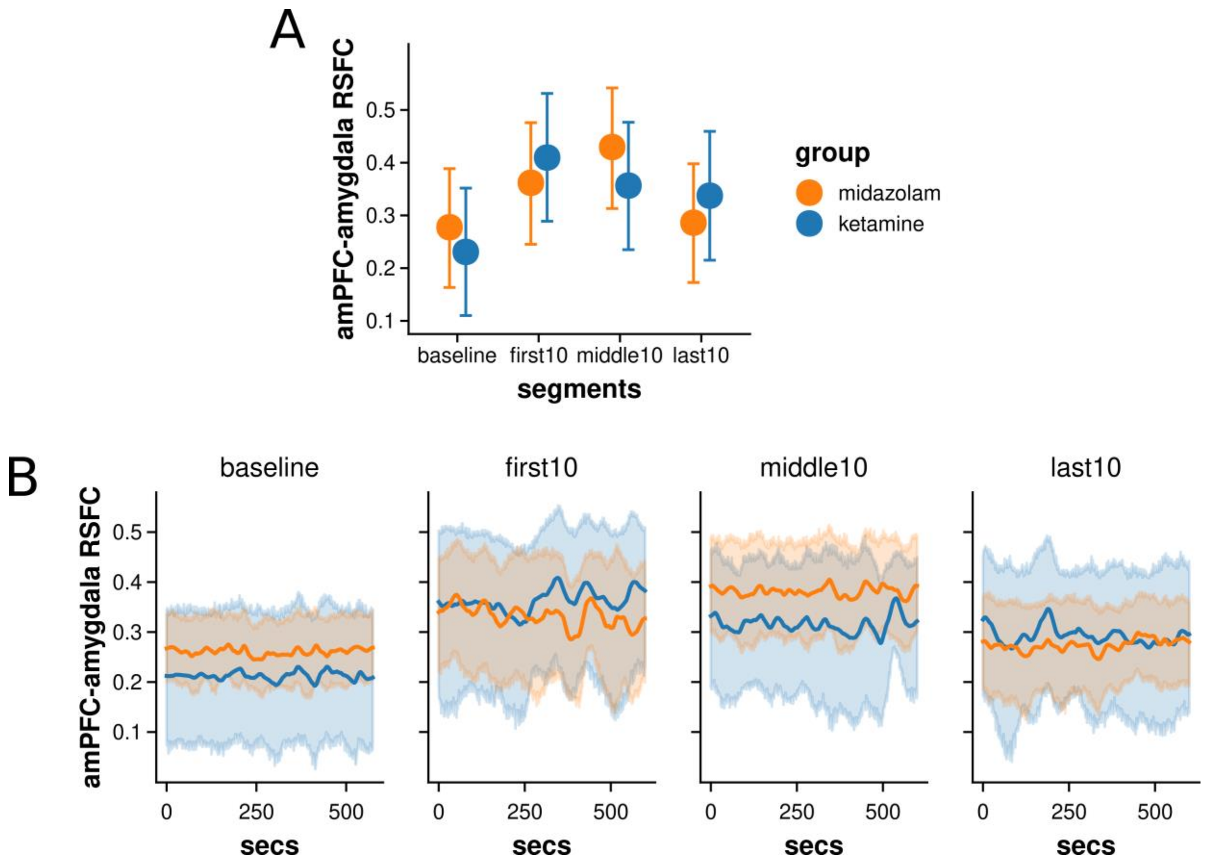

Figure S3. (A) Fitted values of a Bayesian multilevel regression model predicting (static) amPFC-amygdala RSFC by group (ketamine vs. midazolam) and segment (baseline; first, middle, and last 10-minutes of drug infusion). Vertical lines represent 89%CIs. (B) Timepoint-by-timepoint (dynamic) amPFC-amygdala RSFC.
